# Attitudes, Help-Seeking Barriers, and Predictors of Intention to Use Telemental Health Services Among University Students in Saudi Arabia: A Cross-Sectional Study

**DOI:** 10.3390/healthcare14111468

**Published:** 2026-05-26

**Authors:** Yahia Aldhamri

**Affiliations:** Community and Psychiatric Mental Health Nursing Department, College of Nursing, King Saud University, Riyadh 12372, Saudi Arabia; yaldhamri@ksu.edu.sa

**Keywords:** telemental health, university students, Saudi Arabia, Technology Acceptance Model, Theory of Planned Behavior, help-seeking barriers

## Abstract

**Highlights:**

**What are the main findings?**

- University students demonstrated moderately positive attitudes toward telemental health services, with attitude emerging as the strongest predictor of behavioral intention to use these services.
- The lack of trust in mental health professionals was the most prominent help-seeking barrier

**What are the implications of the main findings?**

- Universities and providers should strengthen privacy features and expand telemental health options.
- There is a need for culturally tailored awareness campaigns, trust-building communication, and gender-sensitive service design to support telemental health adoption among Saudi university students.

**Abstract:**

Background: Mental health concerns are notably common among students attending universities in Saudi Arabia, and low engagement with psychological services has been widely documented in this population group. Telemental health has emerged as a promising alternative under Vision 2030’s digital transformation agenda, although the determinants of university students’ intentions to use these services have received limited empirical attention in Saudi Arabia. Objective: This study examined attitudes toward telemental health services, perceived barriers to seeking psychological help, and predictors of behavioral intentions to use telemental health services among university students in Saudi Arabia, based on the Technology Acceptance Model and Theory of Planned Behavior. Methods: A cross-sectional design was employed using an online, self-administered questionnaire. A total of 236 undergraduate students from three large universities in Riyadh were recruited using convenience sampling methods. We examined demographic variables, telemental health attitude variables (ease of use, usefulness, subjective norms, trust in telemental health, relative advantage, intentions, and attitudes), and barrier subscales (fear of stigma, trust in mental health professionals, difficulties in self-disclosure, perceived devaluation, and lack of knowledge) among university students. Descriptive statistics, Welch’s *t*-tests, and multiple linear regression analyses were conducted using SPSS (version 29). Results: Participants demonstrated moderately positive attitudes toward telemental health (M = 74.15, SD = 16.11) and reported moderate overall barriers (M = 50.76, SD = 14.44), with trust in mental health professionals being the most prominent barrier. The regression model explained 58.0% of the variance in behavioral intentions (F(19, 211) = 15.35, *p* < 0.001). Attitude was the strongest predictor (β = 0.534, *p* < 0.001), followed by trust in telemental health, sex, and difficulty in self-disclosure. Conclusions: Culturally tailored awareness campaigns, trust-building communication, and gender-sensitive service design are recommended to promote the adoption of telemental health by Saudi university students. These efforts align with Vision 2030’s digital health priorities and may support the equitable expansion of mental healthcare access in this population.

## 1. Introduction

Public health and higher education in Saudi Arabia have prioritized mental health, particularly among young adults, who face increasing emotional, social, and intellectual demands during their academic journeys. The mental health of university students has become a serious public health concern, with researchers consistently reporting elevated rates of stress, anxiety, and depression in this population [1,2]. Despite the availability of on-campus counseling services and national awareness campaigns, many students remain reluctant to seek professional psychiatric support, and the propensity to seek mental health treatment in Saudi Arabia is consistently low [3,4,5,6].

Telemental health is a subset of telemedicine that refers to the delivery of mental health services through telecommunication technologies, including videoconferencing, mobile devices, e-mail, chat, text, and the Internet [7]. For university students, telemental health offers accessible, private, and convenient support by removing common logistical barriers to mental health care, such as transportation difficulties and scheduling conflicts. It also has the potential to reduce stigma and attitudinal barriers that prevent students from seeking help.

Saudi Arabia has invested heavily in its electronic health infrastructure, as evidenced by the establishment of the Sehha virtual hospital and the Qareboon application, in line with Vision 2030, which prioritizes digital transformation and mental healthcare improvement [8]. Additionally, the COVID-19 pandemic accelerated the adoption and use of telemental health solutions as a safe and practical alternative to in-person consultations globally and in Saudi Arabia. However, the effectiveness of telemental health depends on students’ willingness to use these services, which is shaped by different factors such as perceived ease of use, perceived usefulness, trust in telemental health, and overall attitudes. Nevertheless, the level of acceptance and intention to use telemental health among Saudi university students remains poorly understood. Understanding university students’ attitudes toward telemental health is essential for encouraging positive engagement and reducing barriers to use. Therefore, this study aimed to investigate university students’ intentions toward telemental health utilization and their perceived barriers to seeking psychological health services.

Researchers have investigated the factors influencing people’s attitudes and behavior toward utilizing telemental health via various theoretical frameworks, which have demonstrated their utility in clarifying the determinants of digital mental health service adoption among university students. Two commonly utilized theories to understand students’ acceptance and intentions to use telemental health include the Technology Acceptance Model (TAM) [9] and the Theory of Planned Behavior (TPB) [10]. TAM suggests that students’ willingness to try digital mental health services hinges on whether they find them helpful for their needs (perceived usefulness) and easy to use (perceived ease of use). TPB adds a psychological lens, proposing that intentions are shaped by one’s attitude toward using telemental health services, social influence perceived from others (subjective norms), and confidence in being able to use and access telemental health services (perceived behavioral control). Collectively, these theories employ straightforward language to describe the combination of individual convictions and societal influences that promote the adoption of telemental health solutions. We incorporated TAM with TPB variables to explain university students’ intentions to adopt telemental health services.

Research conducted globally indicates that university students hold positive views of telemental health, although usage rates vary by country. Students in South Africa, Bangladesh, Thailand, China, and Nigeria have demonstrated favorable attitudes toward telemental health [11,12,13,14,15]. In contrast, students in Western countries have expressed concerns about privacy, confidentiality, and trust in telemental health, with some preferring in-person or hybrid interactions [16,17,18,19].

In Saudi Arabia, research on university students’ perceptions of telemental health services is limited. The available evidence indicates broadly positive attitudes toward telemental health [20,21,22]. Students recognized the convenience and technological advantages of telemental health in Saudi Arabia, given the country’s advanced digital infrastructure and widespread smartphone usage. University students attributed positive attitudes to Internet access and cultural influence [22]. However, affordability and trust emerged as barriers, with findings indicating that students tended to share less personal information. Notably, 64% of students preferred to disclose less information in virtual consultations than in in-person interactions [22]. Similarly, Alanazi et al. [20] conducted a study involving 352 patients and 27 psychiatrists and found a strong consensus on the benefits of telepsychiatry. Age was a significant factor influencing attitudes, with younger individuals exhibiting more positive attitudes. In a larger sample, Alumran et al. [21] found that while awareness of mental health applications was relatively common among Saudis, actual usage remained limited, with only one in five participants reporting use. Despite these findings, several areas of telemental health usage in Saudi Arabia remain understudied, particularly the factors that may influence university students’ intentions and use of telemental health services. The current study aimed to (1) examine university students’ attitudes toward telemental health, (2) assess perceived barriers to seeking psychological help, and (3) determine whether attitudinal factors (perceived ease of use, perceived usefulness, subjective norms, trust, relative advantage, and overall attitude) and help-seeking barriers (fear of stigma, distrust in mental health professionals, difficulties in self-disclosure, perceived devaluation, and lack of knowledge) predict intentions to use telemental health services.

## 2. Materials and Methods

### 2.1. Design

This study adopted a cross-sectional descriptive approach, with data collected through an online questionnaire that participants completed independently. Data collection took approximately three months to complete, from October to December 2025. The questionnaire was distributed electronically through university notification systems and social media channels targeting the study population.

### 2.2. Setting and Participants

The study recruited undergraduate students from three major universities located in Riyadh, Saudi Arabia: King Saud University, Princess Nourah Bint Abdulrahman University, and Imam Mohammad Ibn Saud Islamic University. These universities offer an extensive selection of academic programs in various fields. The target population consisted of students pursuing bachelor’s degrees in all academic disciplines. Participants were recruited using non-probability convenience sampling, a method frequently employed due to its practicality and efficiency in accessing a large number of targeted populations.

To ensure sufficient statistical power, the sample size was estimated using the G*Power software (Version 3.1.9.6). The calculation was based on the F test for a fixed-model multiple linear regression testing the deviation of R^2^ from zero. The parameters included 19 predictors, an alpha level of 0.05, a medium effect size (f^2^ = 0.15), and a target statistical power of 0.95. Due to the varied results from previous research on factors affecting the intention and attitudes toward using digital and telemental health services, a medium effect size of f^2^ = 0.15 was chosen [12,13,23,24]. The parameters suggested that a minimum of 213 participants is required. A total of 236 completed responses were obtained, surpassing the required number. The eligibility criteria included university students aged 18 years or older who were enrolled in a bachelor’s degree program. Students diagnosed with a severe mental health condition and those who opted not to participate or did not complete the questionnaire were excluded from the analyses.

### 2.3. Measures

#### 2.3.1. Sociodemographic, Academic, and Familiarity Variables

Sociodemographic and academic data were collected using a structured questionnaire, including sex, age, academic year, field of study (health or non-health), and cumulative grade point average (GPA). Two familiarity variables were also assessed using a single-item measure: previous engagement with mental health services and the presence of family members with mental health disorders, and participants responded to each item with a yes or no answer. The inclusion of these variables aimed to explore potential differences in attitudes and intentions toward telemental health among subgroups with varying degrees of exposure to mental health care services.

#### 2.3.2. Attitude Toward Telemental Health

Attitudes toward telemental health services were assessed using the Attitudes toward telemental health scale developed by Xue et al. [23], which evaluates individuals’ perceptions and attitudes toward telemental health. The instrument comprised 21 items that captures 5 perception domains, along with overall attitude and behavioral intention components. The five perception subscales were perceived usefulness (3 items), perceived ease of use (3 items), subjective norms (2 items), relative advantage (4 items), and trust (3 items), attitude toward telemental health (3 items) and behavioral intention toward utilizing telemental health (3 items). Items are rated on a 5-point Likert-type scale ranging from 1 (strongly disagree) to 5 (strongly agree), with higher scores indicating more favorable attitudes. A representative item is “Using telemental health services would improve the quality of mental health care I receive”. Total scores ranged from 21 to 105, with higher scores indicating more favorable attitudes and a greater propensity to adopt telemental health services. In addition, subscale scores were computed by summing the relevant items, yielding possible ranges of 3 to 15 for perceived usefulness, perceived ease of use, trust, overall attitude, and behavioral intention, 2 to 10 for subjective norms, and 4 to 20 for relative advantage. The instrument was selected because it integrates core TAM constructs with trust and stigma-related factors that are particularly relevant in contexts where reluctance to disclose mental health concerns may influence the adoption of telemental health.

The six perception subscales assess conceptually distinct dimensions that together shape the evaluative orientation toward the telemental health. The perceived usefulness subscale evaluates the extent to which respondents believe that telemental health services would enhance the effectiveness and outcomes of mental health care. The perceived ease of use subscale captures the degree to which engaging with telemental health is viewed as effortless and straightforward. The subjective norms subscale reflects perceived social pressure and the influence of important referents, such as family members, friends, and peers, on the decision to adopt telemental health. The relative advantage subscale assesses the extent to which telemental health is perceived as superior to conventional face-to-face mental health care in terms of convenience, accessibility, and flexibility. The trust subscale evaluates confidence in the reliability, confidentiality, and professional integrity of the telemental health providers and platforms. The fear of stigma subscale assesses the degree to which respondents anticipate social judgment or personal discomfort associated with seeking help through telemental health, with items on this subscale reverse scored so that higher values reflect lower perceived stigma and thus a more favorable orientation toward telemental health. The overall attitude component captures the general evaluative judgment toward telemental health, and the behavioral intention component assesses readiness and willingness to adopt telemental health services. The scale was translated and culturally adapted for use with an Arabic-speaking Saudi population using a standard four-step procedure of forward translation by two bilingual translators, expert panel review for clarity and cultural relevance, back translation to confirm semantic equivalence, and pilot testing with a small group of students. However, the overall scale demonstrated excellent internal consistency (α = 0.95) across all 21 items in the present study. Among the subscales, intentions had the highest reliability (α = 0.95), followed by usefulness (α = 0.93), ease of use (α = 0.86), trust (α = 0.85), attitude (α = 0.82), relative advantage (α = 0.77), and subjective norms (α = 0.76). All subscales met or exceeded the acceptable threshold of 0.70.

#### 2.3.3. Barriers to Psychological Help

Perceived barriers to seeking psychological help were assessed using the Barriers to Seeking Psychological Help Scale for College Students, developed by Topkaya et al. [25]. The instrument consists of 17 items rated on a 5-point Likert-type scale ranging from 1 (strongly disagree) to 5 (strongly agree), with higher scores indicating greater perceived barriers and stronger reluctance to seek psychological help. A representative item is “I worry about being stigmatized as ‘problematic’ and/or ‘crazy’ if I seek psychological help.” The scale was selected because it was developed specifically within a college student population and captures a culturally salient set of barriers that are conceptually aligned with the help-seeking challenges commonly documented among university students in collectivist and culturally conservative societies, including Saudi Arabia.

The Barriers to Seeking Psychological Help Scale comprises five subscales that reflect conceptually distinct categories of barriers. The fear of being stigmatized by society subscale captures concerns about negative social judgment, labeling, and reputational harm that students anticipate if others learn that they have sought psychological help. The trust in the mental health professional subscale reflects doubts about the competence, attentiveness, and responsiveness of traditional mental health professionals, including concerns that the professional may not listen adequately or understand the student’s concerns. The difficulties in self-disclosure subscale assesses the reluctance to share personal, emotional, or private problems with a professional, reflecting discomfort in disclosing sensitive material, even within a confidential clinical setting. The perceived devaluation subscale captures negative self-evaluations that students anticipate as a consequence of seeking help, such as feeling weak or experiencing a decline in self-confidence. The lack of knowledge subscale reflects informational and structural barriers, including uncertainty regarding how to contact mental health professionals and limited awareness of available services. Together, these five dimensions capture personal, sociocultural, and institutional sources of reluctance, consistent with the established conceptualizations of help-seeking barriers in college populations [25].

Barriers to Seeking Psychological Help Scale was also translated and culturally adapted for use with an Arabic-speaking Saudi population following the same four-step procedure described above. In the present study, the overall scale demonstrated excellent internal consistency (α = 0.92) for all 17 items. The fear of stigma subscale had the highest reliability (α = 0.84), followed by trust in mental health professionals (α = 0.83), perceived devaluation (α = 0.81), and lack of knowledge (α = 0.70). However, the difficulty of self-disclosure fell marginally below the conventional threshold (α = 0.67) and is acknowledged as a limitation of this study.

### 2.4. Data Collection

Data were gathered using an online self-administered questionnaire created using Google Forms. The questionnaire included an informed consent page and three sections: sociodemographic and familiarity characteristics, the Attitude Toward Telemental Health Services Scale [23], and the Barriers to Seeking Psychological Help Scale for College Students [25]. Recruitment proceeded by sharing the survey link via social media and the official university’s notification system. The recruitment letter provided a concise summary of the study’s objectives and anticipated duration for completion and emphasized the voluntary nature of participation.

Several measures were taken to ensure that respondents belonged to the target population. The introduction to the survey stated the inclusion criteria clearly, noting that participation was open only to currently enrolled university students. The survey platform was set to allow only one response per device, which prevented duplicate submissions. Recruitment took place through university-affiliated student groups and social media channels, which helped direct the survey toward the intended population.

### 2.5. Data Analysis

Data were imported into IBM SPSS Statistics version 29 for cleaning, coding, and statistical analysis. Descriptive statistics were computed to summarize the sample’s sociodemographic characteristics, attitudes toward telemental health, and perceived barriers, including percentages, frequencies, means, and standard deviations. Internal consistency reliability was assessed using Cronbach’s alpha coefficients for all the scales and subscales. Additionally, Welch’s *t*-tests were conducted to examine differences in attitudes toward telemental health services and barriers to seeking psychological help across demographic and familiarity variables (sex, field of study, having a family member with mental illness, and previous mental health utilization). Welch’s *t*-test was selected to account for potential inequality of variances between groups, as it does not assume homogeneity of variance. Before interpreting the findings, normality was evaluated by inspecting the histograms of each group, which indicated approximately normal distributions. Effect sizes were estimated using Cohen’s d, with values of 0.20, 0.50, and 0.80 interpreted as small, medium, and large, respectively [26].

Furthermore, a multiple linear regression analysis was also performed to examine predictors of behavioral intentions to use telemental health services. The regression model included demographic variables, telemental health attitude subscales (ease of use, usefulness, subjective norms, trust, relative advantage, and overall attitude), and barrier subscales (fear of stigma, trust in mental health professionals, difficulties in self-disclosure, perceived devaluation, and lack of knowledge). The reference categories were as follows: health field for study field, male for sex, single for marital status, students in the first through fourth academic years for academic level, yes for previous mental health service use, and yes for having a relative with a mental illness. Regression assumptions were evaluated prior to model interpretation. Normality of residuals was assessed by inspecting the histogram and Q-Q plot of standardized residuals, homoscedasticity was examined using a scatterplot of standardized residuals against predicted values, and multicollinearity was evaluated using tolerance values (>0.1) and variance inflation factor statistics (<10). Influential cases were identified using Cook’s distance, and no cases exceeded the recommended threshold. No violations of assumptions were detected. The significance was determined by a *p*-value threshold of less than 0.05.

### 2.6. Ethical Considerations

The Institutional Review Board at King Saud University granted ethical approval for this study (KSU-HE-25-116; 2 February 2025). All procedures were performed in accordance with relevant guidelines and regulations, including the principles outlined in the Declaration of Helsinki. All participants provided electronic informed consent, and the consent form clearly outlined the study’s objectives, methods, potential risks, and benefits. To ensure confidentiality and anonymity, no personally identifiable information was collected, and access to the dataset was restricted to the research team.

## 3. Results

### 3.1. Participants’ Sociodemographic, Academic, and Familiarity Variables

The study involved 236 university students. The mean age was 21.52 years (SD = 2.03; range 18–27), and the mean GPA (out of 5) was 4.24 out of 5.0 (SD = 0.50). The majority were female (*n* = 140, 59.3%) and single (*n* = 230, 97.5%). Students in the internship or training phase represented the largest group (*n* = 88, 37.3%), followed by fourth-year students (*n* = 55, 23.3%). Most participants were enrolled in health-related fields (*n* = 164, 69.5%), and nearly half reported having a relative with a mental illness (*n* = 110, 46.6%). The demographic, academic, and familiarity variables of the participants are summarized in Table 1.

### 3.2. Attitudes Toward Telemental Health Services

The overall attitude toward telemental health had a mean of 74.15 (SD = 16.11), indicating moderately positive attitudes toward telemental health. The relative advantage subscale recorded the highest mean (M = 13.82, SD = 3.33), followed by ease of use (M = 11.24, SD = 2.80), intention (M = 11.04, SD = 3.34), attitude (M = 10.94, SD = 2.78), usefulness (M = 10.83, SD = 3.03), and trust (M = 10.71, SD = 2.83) subscales. The reported mean of the intentions subscale suggested moderate to high intentions to use telemental health services and favorable attitudes toward telemental health by university students. The subjective norms subscale had the lowest mean (M = 5.55, SD = 2.12), indicating that participants perceived limited social influence encouraging the use of telemental health. The descriptive statistics are presented in Table 2.

### 3.3. Barriers to Seeking Psychological Health

The overall barrier score had a mean of 50.76 (SD = 14.44), indicating a moderate level of perceived barriers to seeking psychological help. Trust in traditional mental health professionals recorded the highest mean (M = 13.35, SD = 4.22), falling within the high-barrier range according to the scale’s interpretation guidelines, whereas fear of stigma (M = 10.69, SD = 4.38), self-disclosure (M = 9.28, SD = 3.06), lack of knowledge (M = 8.94, SD = 3.00), and perceived devaluation (M = 8.49, SD = 3.38) were within the moderate range (Table 3).

### 3.4. Group Differences in Attitudes Toward Telemental Health Services and Perceived Barriers to Seeking Psychological Help

Welch’s *t*-tests were conducted to examine whether attitudes toward telemental health services and barriers to seeking psychological help differed across sex, field of study, having a relative with a mental illness, and previous mental health utilization. No significant differences were found in overall telemental health attitudes or any of the six attitude subscales across all demographic variables (*p* > 0.05), indicating consistently similar attitudes among participants. In contrast, several significant differences emerged in the barriers to seeking psychological help (Table 4). Male students reported significantly higher fear of stigma than female students (t = 2.412, *p* = 0.017, d = 0.31). Female students reported significantly higher distrust of traditional mental health professionals than male students (t = −2.79, *p* = 0.006, d = 0.37). Additionally, non-health field students demonstrated a significantly greater distrust toward traditional mental health professionals than health field students (t = −2.73, *p* = 0.007, d = 0.37). No other barrier subscales or the overall barriers composite demonstrated statistically significant differences across the examined demographic variables (*p* > 0.05).

### 3.5. Predictors of Intentions to Use Telemental Health

A multiple linear regression was conducted to identify predictors of behavioral intentions toward utilizing telemental health services. Diagnostic assumptions, including normality of residuals, linearity, homoscedasticity, and multicollinearity, were evaluated, and no violations were detected. The regression model included demographic and familiarity variables, telemental health attitudinal subscales, and psychological help-seeking barrier subscales.

The regression model was statistically significant, F(19, 211) = 15.35, *p* < 0.001, and explained 58.0% of the variance in behavioral intentions (R^2^ = 0.580, adjusted R^2^ = 0.543). Four predictors were found to be statistically significant. Among the subscales of attitudes toward telemental health, attitude emerged as the strongest predictor (β = 0.638, *p* < 0.001), followed by sex (β = 0.763, *p* = 0.023), with female students reporting higher intentions, trust in telemental health (β = 0.198, *p* = 0.035), and difficulties in self-disclosure (β = −0.166, *p* = 0.020). The regression coefficients are listed in Table 5.

## 4. Discussion

This study examined the attitudes, perceived barriers to seeking psychological help, and predictors of intention to use telemental health among university students in Saudi Arabia. We found that students held moderately positive attitudes toward telemental health and reported moderate overall barriers, with trust in mental health professionals being a prominent barrier. Our findings also indicate that attitudes toward telemental health were consistent across groups, suggesting broad acceptance. Nevertheless, differences in barriers suggest that stigma may affect male students more, while mistrust toward professionals is higher among female and non-health students. Attitude, trust in telemental health, difficulties in self-disclosure, and sex predicted behavioral intentions toward the use of telemental health services.

The moderately positive attitudes observed in this study are consistent with prior findings among university students globally [11,12,13,14,15,20,21,22,27]. In Saudi Arabia, Aldekhyyel et al. [27] reported that 82% of Saudi health consumers viewed telemedicine as efficient, economical, and capable of providing access to specialized care. The positive attitude we observed can be attributed to participants’ favorable perceptions of ease of use, relative advantage over in-person services, and trust in remote service quality. The advanced digital infrastructure and widespread smartphone usage in Saudi Arabia, together with Vision 2030 investments in e-health, may have created an environment in which technology-based mental health services are familiar and acceptable [8,22]. However, the relatively low subjective norms score may reflect the cultural context in which mental health remains a sensitive topic and discussions about seeking telemental health support may be avoided [22,28,29], leaving students with few role models or social influences encouraging them to openly endorse telemental health use. Additionally, Bugis et al. [22] reported that nearly 45% of university students in Saudi Arabia had never engaged with any form of telemental health service. This limited exposure suggests that the broader social environment has not fostered sufficient awareness or normalization of such services. Consequently, mechanisms such as peer influence and social endorsement remain underdeveloped and do not significantly shape individuals’ intentions to use telemental health.

Our findings indicate that university students reported moderate overall barriers to seeking psychological help. Trust in mental health professionals was rated as the most significant barrier to help-seeking. This finding can be attributed to the broader cultural attitudes toward mental health professionals in Saudi society, where the workforce is still developing, and public familiarity with mental health services remains limited to government platforms such as the Qareboon application [21]. Furthermore, our findings align with those of Bugis et al. [22] who reported that 64% of Saudi students preferred disclosing less information in virtual consultations than in-person interactions. Aldhamri et al. [30] suggested that university students tend not to consider mental health specialists as their first source of help for mental health issues. Nevertheless, fear of stigma, perceived devaluation, and lack of knowledge fell in the moderate range of the scale’s interpretation guideline, suggesting that these barriers exist but are less prominent than trust and self-disclosure concerns in shaping intentions to use telemental health services. Notably, our findings revealed that attitudes toward telemental health services were similar across the demographic groups. In contrast, the barriers students face when seeking psychological help vary by sex and field of study. This suggests that attitudes toward telemental health may be shaped more by technology beliefs, which are widely shared within the student population.

The strength of the attitude effect suggests that Saudi students who appraise telemental health as beneficial are highly motivated to engage with such services in the future. This finding is consistent with the TPB, which posits attitude as a proximal determinant of intention [10], and with previous research in which attitude was the strongest predictor of digital health platform use among university students [12,13,23,24]. The consistency of attitude as the strongest predictor across these studies reinforces the central role that attitude plays in technology acceptance. However, the relatively high explained variance observed in our study can be attributed to the fact that we included a comprehensive set of predictors that combined TAM constructs (ease of use, usefulness, subjective norms, trust, relative advantage, and attitude) with barriers to psychological help-seeking and demographic variables, which captured both technology-related and psychological factors that influence intention.

In our study, trust in telemental health predicted the behavioral intentions. This finding highlights the importance of trust in shaping the willingness to use telemental health, which aligns with research showing that Saudi users express concerns about data privacy and confidentiality on digital platforms and prefer to share less personal information in virtual settings [22,28]. This finding demonstrates that trust is essential for adopting telemental health in culturally conservative countries. In Saudi Arabia, where telemental health is still in its early stages, fostering trust in these services is crucial for increasing their use.

Within the integrated TPB and TAM framework, our findings support the view that technology-related beliefs (perceived usefulness, ease of use, and trust in the platform) and help-seeking-related beliefs (stigma, provider trust, and self-disclosure comfort) operate via separate pathways. From a practical perspective, this implies that promoting telemental health requires a dual approach. It involves maintaining favorable attitudes through awareness campaigns and platform quality, while also addressing the differential barriers that may prevent certain groups from translating these attitudes into actual help-seeking behaviors.

Among the barrier subscales, difficulties in self-disclosure significantly predicted lower intention. This indicates that students who anticipate discomfort sharing personal information with professionals are less likely to use telemental health services. This finding aligns with Saudi research showing that individuals tend to keep mental health concerns private to avoid social repercussions for themselves and their families [3,28,29]. A possible explanation is that university students often lack a private physical space in which to engage with telemental health, as documented in previous studies [17,31]. The non-significance of fear of stigma, perceived devaluation, and lack of knowledge as predictors suggests that these traditional help-seeking barriers may operate differently in technology-mediated care than in face-to-face services. Future research should investigate these nonsignificant barriers further and examine their roles in predicting telemental health care usage.

Sex was a significant predictor, with female students reporting higher intentions of using telemental health services. This finding can be explained by the cultural context in Saudi Arabia, where remote modalities may help reduce the social and logistical constraints that women face when seeking in-person mental health care [21,28]. This finding has been documented in both Saudi and international literature [15,21,22,28,30,32]. In contrast, age was not a significant predictor in our sample, which differs from the findings of Alanazi et al. [20], who found more favorable attitudes among younger Saudi adults. This difference may be due to the characteristics of the sample. We included only undergraduate students, resulting in a narrow age range for our sample. In contrast, their study included the general Saudi population across a wider age range.

Regarding the differences among participants, differences in barriers suggest that stigma may affect male students more, while mistrust toward mental health professionals appears higher among female and non-health students. The higher stigma among male students may be indicative of the impact of masculinity norms within Saudi culture (Mostoller et al., 2024), male students may view emotional openness and psychological struggles as conflicting with their sense of male identity, which can discourage them from seeking assistance [29,33,34]. Furthermore, the observed sex difference suggests that female students may have greater reservations about mental health professionals than male students. Aldaweesh et al. [28] reported that Saudi women sometimes prefer commercial and private sector mental health apps when seeking help because they offer anonymity, distance, and more control over personal information. The observed difference is important when considered alongside the regression results of the current study. Although male students’ attitudes toward telemental health were positive, stigmatizing attitudes may prevent them from seeking help for mental health issues. Additionally, telemental health may be more appealing to female students who are cautious about traditional face-to-face providers, as the digital format provides a sense of privacy and protection from potential predators. Therefore, interventions targeting male university students should address stigma by employing messaging that normalizes mental health issues and presents telemental health as compatible with masculine self-reliance and privacy issues.

Four of the five barrier subscales (fear of stigma, trust in mental health professionals, perceived devaluation, and lack of knowledge) did not shape the willingness to use telemental health, indicating that barriers typically associated with traditional psychological help-seeking may not operate similarly in the context of telemental health. Factors such as stigma, distrust of professionals, and perceived devaluation are often rooted in direct interpersonal interactions. In contrast, telemental health services can mitigate these concerns by providing greater anonymity, physical separation, and reduced social visibility, thereby altering the experience of these barriers.

Given that attitude was the strongest predictor of intention, universities in Saudi Arabia should implement educational programs that introduce students to telemental health and demonstrate its benefits and effectiveness. The central role of trust suggests that providers should prioritize transparency, demonstrate clinical competence, and communicate platform security clearly to potential users. The negative effect of self-disclosure barriers suggests that platforms should offer privacy-preserving features and graduated engagement options. The higher intentions among female students indicate that telemental health may be a particularly important avenue for expanding mental health access for Saudi women, who continue to face additional cultural barriers to in-person care.

Our study had several limitations. First, the cross-sectional design does not allow for causal inferences; longitudinal designs are needed to examine how attitudes and intentions change over time and translate into actual use of telemental health. Second, the study relied on self-reported measures, which may be subject to social desirability bias, particularly in a cultural context where mental health topics are sensitive issues. Third, the sample was drawn from three universities in Riyadh and may not be generalizable to the broader Saudi student population. The convenience sampling approach further limits external validity. Additionally, we did not record the number of participants per university, which prevents any assessment of whether specific institutions were over-represented or whether participation was balanced across sites. Finally, the difficulties in self-disclosure subscale showed weak internal consistency; therefore, the findings should be interpreted cautiously. Future research should examine the role of digital and eHealth literacy, social influence from peers, prior experience with telehealth, and culturally specific values such as collectivism and family interdependence in shaping telemental health adoption among Saudi university students. Additionally, future researchers could utilize longitudinal or mixed-method designs to gain a deeper understanding of the acceptance and attitudes of university students toward telemental health services in Saudi Arabia. They can also consider record institutional affiliation which would allow direct comparison across institutions and a clearer evaluation of representativeness and site-level differences in attitudes toward telemental health services.

## 5. Conclusions

This study examined the attitudes, barriers, and predictors of the intention to use telemental health among Saudi university students. Students reported moderately positive attitudes and moderate barriers to seeking help, with a lack of trust in mental health professionals being the main barrier. Attitude was the strongest predictor of intention, followed by trust in telemental health, sex, and difficulty in self-disclosure. The findings show that expanding telemental health in Saudi Arabia requires more than building infrastructure. Universities and providers need to raise awareness to strengthen positive attitudes toward telemental health. They should also be transparent to build trust in digital platforms. Strong privacy features can help reduce concerns regarding the sharing of personal information. Female students showed a greater willingness to use these services, suggesting an opportunity to improve access for women who face cultural barriers to in-person care. In contrast, male students may require targeted support to overcome stigma and seek assistance. Overall, effective implementation requires a culturally sensitive, trust-centered, and gender-responsive approach. These efforts support the goals outlined in the Saudi Vision 2030. Future research should track how attitudes translate into actual use and explore how cultural values shape long-term engagement.

## Figures and Tables

**Table 1:** Sociodemographic and academic characteristics of the participants.

Variable		N	Mean (SD)/Percentage
Age		236	21.52 (2.03)
Grade Point Average		231	4.24 (0.50)
Sex	Male	96	40.7%
	Female	140	59.3%
Marital status	Single	230	97.5%
	Married	6	2.5%
Academic level	1st year	32	13.6%
	2nd year	26	11.0%
	3rd year	35	14.8%
	4th year	55	23.3%
	Internship/training	88	37.3%
Field of study	Health field	164	69.5%
	Non-health field	72	30.5%
Previous mental health use	Yes	55	23.3%
	No	181	76.7%
Relatives with mental illness	Yes	110	46.6%
	No	126	53.4%

SD, standard deviation.

**Table 2:** Descriptive statistics for the attitude toward telemental health scale and subscales (N = 236).

Scale/Subscale	Items	M (SD)	α
Overall Attitude	21	74.14 (16.11)	0.947
Ease of Use	3	11.24 (2.80)	0.862
Usefulness	3	10.83 (3.02)	0.925
Subjective Norms	2	5.55 (2.12)	0.755
Trust in telemental	3	10.71 (2.82)	0.850
Relative Advantage	4	13.82 (3.33)	0.767
Attitude	3	10.94 (2.77)	0.815
Intentions	3	11.03 (3.33)	0.946

Note: M = Mean; SD = Standard Deviation; α = Cronbach’s alpha.

**Table 3:** Descriptive statistics for the barriers to seeking psychological help scale and subscales (N = 236).

Scale/Subscale	Items	M (SD)	α
Overall, Barriers	17	50.75 (14.44)	0.915
Fear of Stigma	4	10.69 (4.379)	0.841
Trust in MHP	4	13.35 (4.22)	0.834
Self-disclosure	3	9.28 (3.06)	0.675
Perceived Devaluation	3	8.48 (3.37)	0.812
Lack of Knowledge	3	8.94 (3.00)	0.696

Note: M = Mean; SD = Standard Deviation. MHP: Mental Health Professionals.

**Table 4:** Differences in barriers to seeking psychological help based on significant demographic characteristics (N = 236).

Characteristics	*n*	Fear of Stigma				Distrust of MHP ^a^			
		M	SD	t (df) ^b^	*p*	M	SD	t (df)	*p*
Sex									
Male	96	11.50	4.04	2.41 (218.2)	0.017 *	12.44	4.20	−2.79 (202.1)	0.006 **
Female	140	10.14	4.53			13.98	4.13		
Study Field									
Health	164	10.71	4.15	0.09	0.922	12.87	4.24	−2.73 (143.6)	0.007 **
Non-Health	72	10.65	4.89			14.44	3.99		
Previous Mental Health Use									
Yes	55	10.58	4.13	−0.21	0.827	13.69	4.04	0.68	0.497
No	181	10.73	4.46			13.25	4.28		
Relatives with Mental Illness									
Yes	110	10.44	4.66	−0.84	0.398	110	13.46	4.44	0.380
No	126	10.92	4.12			126	13.25	4.04	

Note: ^a^ MHP = mental health professionals; ^b^ df = degrees of freedom. * *p* < 0.05; ** *p* < 0.01.

**Table 5:** Multiple linear regression analysis predicting the intentions to use telemental health services among Saudi university students (*N* = 231).

Predictor	B^a^	SE	β^b^	t	*p*-Value	95% CI (Lower, Upper)
Age	0.010	0.096	0.006	0.108	0.914	−0.179, 0.200
Field of study ^a^	0.081	0.365	0.011	0.223	0.824	−0.638, 0.801
Sex ^b^	0.763	0.333	0.113	2.295	0.023 *	0.108, 1.419
Marital status ^c^	0.310	0.996	0.015	0.311	0.756	−1.655, 2.274
Academic level ^d^	−0.036	0.139	−0.016	−0.257	0.797	−0.311, 0.239
GPA	0.082	0.277	0.014	0.297	0.767	−0.464, 0.628
Previous mental health use ^e^	−0.247	0.375	−0.031	−0.658	0.511	−0.985, 0.492
Relatives with mental illness ^f^	−0.130	0.306	−0.020	−0.425	0.671	−0.733, 0.473
Ease of use	−0.071	0.080	−0.060	−0.888	0.376	−0.230, 0.087
Usefulness	0.118	0.099	0.108	1.199	0.232	−0.076, 0.313
Subjective norms	0.091	0.084	0.057	1.077	0.283	−0.075, 0.256
Trust in telemental	0.198	0.093	0.168	2.121	0.035 *	0.014, 0.382
Relative advantage	−0.025	0.066	−0.025	−0.387	0.699	−0.155, 0.104
Attitude	0.638	0.095	0.534	6.732	<0.001 ***	0.451, 0.824
Fear of stigma	0.005	0.050	0.006	0.091	0.928	−0.095, 0.104
Trust in MHP	0.061	0.054	0.078	1.139	0.256	−0.045, 0.167
Self-disclosure	−0.166	0.071	−0.152	−2.337	0.020 *	−0.306, −0.026
Perceived devaluation	−0.090	0.072	−0.092	−1.256	0.210	−0.231, 0.051
Lack of knowledge	0.049	0.071	0.044	0.691	0.490	−0.091, 0.190
Model summary: R^2^ = 0.580, Adjusted R^2^ = 0.543, F(19, 211) = 15.353, *p* < 0.001						

B^a^, unstandardized coefficient; β^b^, standardized coefficient (beta); CI, confidence interval; GPA, Grade Point Average; * *p* < 0.05; *** *p* < 0.001. ^a^ study field = health field vs. non-health field; ^b^ sex = male vs. female; ^c^ marital status = single vs. married; ^d^ academic levels = students in internship period vs. students in first through fourth years; ^e^ previous mental health use = yes vs. no; ^f^ relatives with mental illness = yes vs. no.

## Data Availability

The data presented in this study are available on request from the author (ylahdmari@ksu.edu.sa) due to privacy and ethical restrictions.

## Abbreviations

The following abbreviations are used in this manuscript:

TAM	Technology Acceptance Model
TPB	Theory of Planned Behavior
GPA	Grade Point Average
MHP	Mental Health Professionals
